# Association of Major Depressive Disorder in Hyperparathyroidism: A Systematic Review

**DOI:** 10.7759/cureus.40150

**Published:** 2023-06-08

**Authors:** Anjali Desai, Anjana Bajgain, Asna Ali, Chandrani Dutta, Khadija Pasha, Salomi Paul, Muhammad S Abbas, Sondos T Nassar, Tasniem Tasha, Safeera Khan

**Affiliations:** 1 Internal Medicine, California Institute of Behavioral Neurosciences & Psychology, Fairfield, USA

**Keywords:** major depressive disorder (mdd), hyperparathyroid, depression, hyperparathyroid-induced hypercalcemia, parathyroid pathology

## Abstract

Major depressive disorder (MDD) is a common neuropsychiatry manifestation that is more prevalent lately. Many contributing factors are present (for example, neurochemical, physiological, pathophysiological, and endocrinological factors). Patients with increased serum parathyroid levels are usually linked to psychosis symptoms but not to depressive symptoms. We conducted this systematic review to explore a correlation between depressive disorder and increased serum parathyroid levels, a major endocrinological pathology, and help establish mental wellness in patients suffering from hyperparathyroidism.

We conducted a thorough literature search using five major databases, MEDLINE, PubMed, PubMed Central (PMC), ScienceDirect, and Google Scholar, using three keywords-MDD, depression, and hyperparathyroidism. We included mixed method studies, including observational studies, non-randomized controlled trials, case reports, and review articles published in the last ten years, focusing on the adult and geriatric population (>18 years) and on depressive and anxiety symptoms associated with patients with hyperparathyroidism. We included 11 articles (seven observational studies + four case reports) for qualitative synthesis after screening the literature. The reviewed studies showed an association between high serum parathyroid level, high serum calcium level, high serum alkaline phosphatase level, low serum phosphorous level, and increased depressive neurocognitive symptoms. After a patient with hyperparathyroidism is treated for hypercalcemia or undergoes parathyroidectomy and the serum parathyroid levels are lowered, a decrease in severe depressive symptoms is noted. The qualitative analysis of the reviewed literature showed an association between major depressive disorder and hyperparathyroidism. This paper can guide clinicians to assess patients with increased serum parathyroid levels for depressive neuropsychiatric symptoms and plan treatment, as treatment of their hyperparathyroidism can significantly lower their depressive symptoms. More randomized controlled trials should be conducted to find the treatment effectiveness of depression in patients with hyperparathyroidism.

## Introduction and background

Parathyroid glands are small glands found on the back of the thyroid gland. They can also be found in the thyroid parenchyma, thymus, pericardium, and anterior or posterior mediastinum [1]. Parathyroid glands secrete parathyroid hormone (PTH), which is mainly regulated by calcium metabolism. Elevated serum calcium (Ca^2+^) levels decrease the release of PTH and vice versa [2]. Primary hyperparathyroidism (PHPT) is a disorder of PTH hypersecretion by parathyroid glands in patients with normal renal function, resulting in increased serum calcium concentration. Secondary hyperparathyroidism mainly occurs with abnormal renal function, vitamin D deficiency, and various endocrinologic disorders [3].

Patients suffering from asymptomatic hyperparathyroidism may display neuropsychiatric symptoms like behavioral disturbances and psychosis, and reduced neurocognitive function [4]. In recent years, many published studies have demonstrated the occurrence of depressive and anxiety disorders in PHPT patients. Most of these studies focused on evaluating the severity of these disorders in patients before and after parathyroidectomy. The results generally indicate a higher prevalence of depressive and anxiety disorders in PHPT patients and alleviation of symptoms postoperatively. Still, the correlation of the pathophysiology of these mental disorders in PHPT patients remains unclear. Rare studies show there is a correlation between the severity of anxiety disorders (severity of neuropsychological symptoms) and the level of PTH (level of calcium ions in serum) [1].

In this systemic review, we aim to explore the association of diagnosed major depressive disorder (MDD) in patients with hyperparathyroidism. We try to explore related pathophysiology and how parathyroidectomy improves depressive symptoms, giving us the answer we are looking for: Is MDD associated with patients with hyperparathyroidism?

## Review

Methods

We used the Preferred Reporting Items for Systemic Reviews and Meta-Analyses (PRISMA 2020) guidelines and principles for this systematic review and reported the results [5].

*Search Sources and Search Strategy* 

Major research literature databases and search engines such as MEDLINE, PubMed, PubMed Central (PMC), ScienceDirect, and Google Scholar were used to search appropriate keywords and Medical Subject Headings (MeSH) thesaurus and find relevant articles about the topic.

The final combined MeSH strategy for PubMed, PMC, and MEDLINE are as follows: MDD OR Depression AND Hyperparathyroidism ("Hyperparathyroidism"[Majr]) AND ("Depression"[Majr]) OR ( "Depression/diagnosis"[Mesh] OR "Depression/mortality"[Mesh] OR "Depression/pathology"[Mesh] OR "Depression/physiology"[Mesh] OR "Depression/physiopathology"[Mesh] OR "Depression/psychology"[Mesh] ) AND ( "Hyperparathyroidism/complications"[Majr] OR "Hyperparathyroidism/diagnosis"[Majr] OR "Hyperparathyroidism/mortality"[Majr] OR "Hyperparathyroidism/pathology"[Majr] OR "Hyperparathyroidism/physiology"[Majr] OR "Hyperparathyroidism/physiopathology"[Majr] OR "Hyperparathyroidism/psychology"[Majr] ).

The keywords used for search in ScienceDirect and Google Scholar included "MDD", "Depression," and "Hyperparathyroidism," to find relevant articles. These keywords were combined in varying combinations using Boolean "AND," "OR," and "NOT."

Inclusion and Exclusion Criteria

We included observational studies, non-randomized controlled trials, case reports, and review articles published in the English language in the last 10 years, focusing on the adult and geriatric population (>18 years) and relevant to our research question. We excluded articles focusing on the pediatric population (<18 years), letters, expert opinions, animal studies, unpublished or grey literature, and papers in languages other than English.

Analysis of Study Quality/Bias

We critically evaluated 15 selected studies for quality using standardized quality assessment tools, and 11 studies qualified as medium or high quality, which were included in the review. The following tools were used:

(a) Newcastle-Ottawa Scale for observational studies [6]

(b) For systematic reviews and meta-analyses, we used the Assessment of Multiple Systematic Reviews (AMSTAR) tool [7]

(c) Scale for the Assessment of Narrative Review Articles (SANRA) checklist for narrative/traditional reviews [8]

(d) The Joanna Briggs Institute (JBI) check tool for case reports [9].

The detailed overall scores and quality for each study are provided in Table 1 and Table 2.

**Table 1:** Summary of the Newcastle-Ottawa risk-of-bias tool for observational studies A quality check was done per the Newcastle-Ottawa Scale [6] (1, 0, N/A). N/A, not applicable Quality: >7/9 = HIGH, >5/9 = MEDIUM, <6/9 = LOW

Studies	Representativeness of exposed cohort	Selection of a nonexposed cohort	Ascertainment of exposure	Demonstration that outcome of interest was not present at the start of the study	Comparability	Study controls for the most important factor (age)	Study controls for any additional factor(s)	Assessment of outcomes	Length of follow up	Adequacy of follow up	Total score	Quality
Kunert et al. [1]	1	1	1	1		1	1	1	N/A	N/A	9/9	HIGH
Duskin et al. [10]	1	1	1	1		1	1	1	N/A	N/A	9/9	HIGH
Wang et al. [2]	1	0	1	1		0	0	1	N/A	N/A	6/9	MEDIUM
Hermsen et al. [3]	1	0	1	1		0	0	1	1	1	7/9	MEDIUM
Zoncco et al. [11]	1	1	1	1		1	1	1	0	0	7/9	MEDIUM
Lui et al. [12]	1	1	1	1		1	1	1	0	0	7/9	MEDIUM
Weber et al. [13]	1	1	1	1		1	1	1	1	1	9/9	HIGH

**Table 2:** Summary of the JBI (Joanna Briggs Institute) check tool for case reports Quality check was done as per the JBI checklist [9] (Y-yes, N-no, N/A). N/A = not applicable

	Grønli et al. [4]	Barrios et al. [14]	Lopes et al. [15]	Sołtysik et al. [16]
Clear history and with the timeline?	Y	Y	Y	Y
The current condition is described as?	Y	Y	Y	Y
Assessment tool?	Y	N	Y	Y
Intervention/procedure described?	Y	Y	Y	Y
Postintervention described?	Y	Y	Y	Y
The adverse event identified as?	Y	N	N	N
Takeaway lessons?	Y	Y	Y	Y
STUDY INCLUDED?	YES	YES	YES	YES

Results

**Figure 1:**
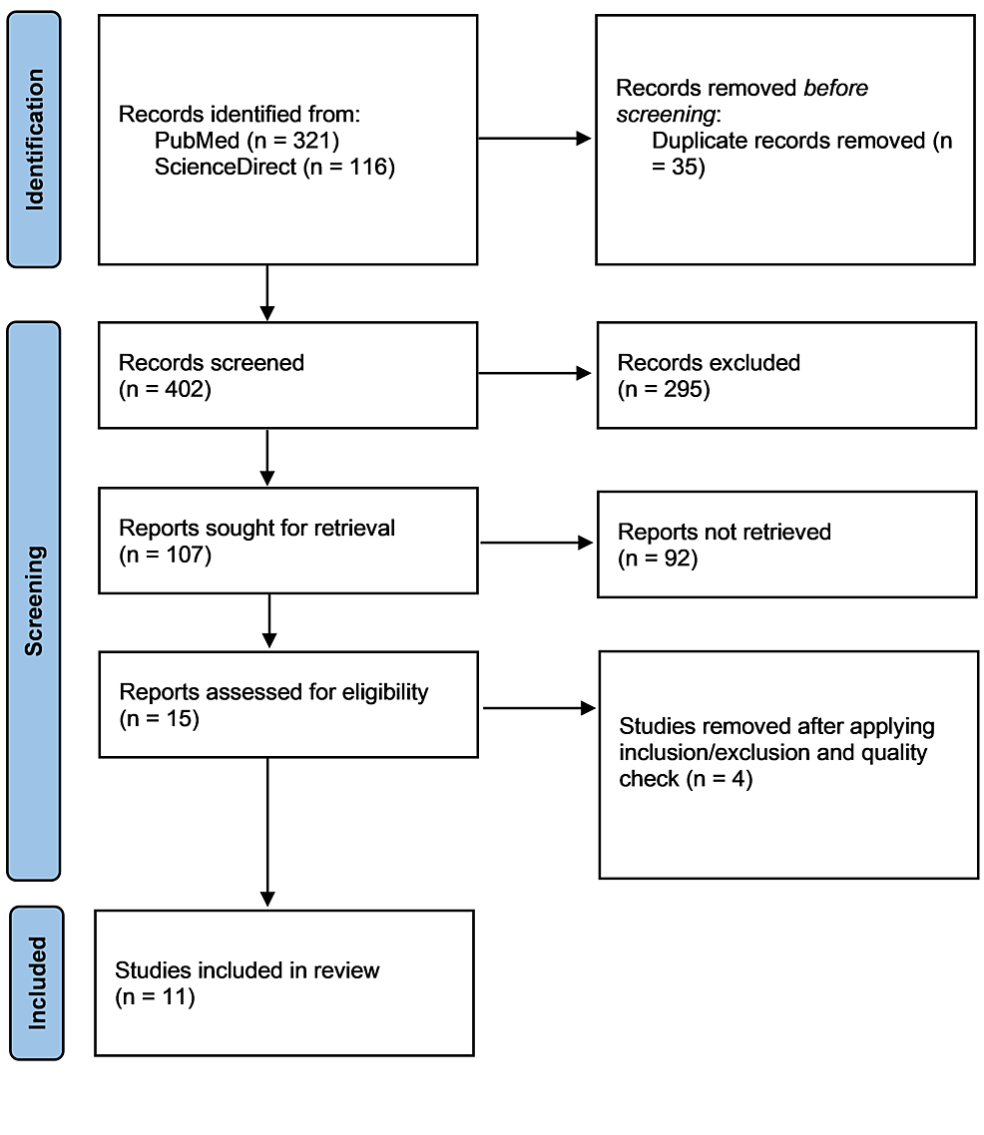
The PRISMA flow chart 2020 (PRISMA = Preferred Reporting Items for Systemic Reviews and Meta-Analyses, n = number of studies, PubMed = PubMed database, ScienceDirect = ScienceDirect database.)

Three hundred seventy-seven articles were identified in our initial search of MEDLINE, PubMed, and PMC databases. Out of them, 321 articles were discarded after applying relevant filters as per our eligibility criteria (last 10 years, human studies), and duplicates were removed. Also, a total of 116 articles were gathered from our initial search of the ScienceDirect database. Fifty-one articles were left after we applied the same eligibility criteria. Two individual investigators then screened the remaining articles (n=107) based on titles, abstracts, full-text, and detailed inclusion-exclusion criteria. After the meticulous screening, application of our inclusion criteria-which were observational studies, non-randomized controlled trials, case reports, and review articles published in the English language in the last 10 years, focusing on the adult and geriatric population (>18 years) and including papers which were relevant to our research question-we were left with 15 articles about our research question. A total of 15 studies were included for a thorough quality/bias assessment using standardized quality assessment tools. Four studies were excluded after quality appraisal, and the final 11 studies were included in this systematic review. We included seven observational studies, out of which four are high quality, and three are medium quality assessed by the Newcastle-Ottawa risk-of-bias tool [6] for observational studies. And we assessed four case reports by the JBI check tool [9], which satisfied the requirements. The PRISMA 2020 flow diagram is depicted in Figure 1 [5].

We included quality-assessed seven observational studies and four case reports for this systemic review. Two case studies focused on the improvement of neurocognitive symptoms after parathyroidectomy whereas two case studies focused on the pathophysiology of psychiatric symptoms in patients with hyperparathyroidism. Of the seven observational studies, four focused on improving depression after surgery in hyperparathyroidism, and three focused on the pathophysiology of calcium levels and parathyroid levels on patients' neurocognitive symptoms.

Our systematic review collectively explored the results of 1227 patients from the reviewed published studies. Out of 1227, 833 patients suffered from hyperparathyroidism, and the remaining were from control groups. Out of 1227, 499 patients were reviewed for the improvement of neuropsychiatric symptoms after parathyroidectomy and 334 patients were reviewed for the effects of increased PTH on their neurocognitive behaviors.

High serum calcium levels and high serum parathyroid levels increase depressive neurocognitive symptoms. The MDD symptoms are also noted with high serum alkaline phosphatase (ALP) and low serum phosphorous levels in patients with hyperparathyroidism. Once treated for hypercalcemia in hyperparathyroidism, the patient's severe depressive symptoms are decreased or under-controlled with anti-depressive medications. Surgery of parathyroid gland removal (parathyroidectomy) lower serum parathyroid levels, serum calcium, and serum ALP level which can be a turnaround in neuropsychiatric depressive symptoms observed in hyperparathyroidism patients. So, the prevalence and severity of MDD decrease if patients with hyperparathyroidism are treated and their serum parathyroid level and the serum calcium level is normalized. 

Discussion

We reviewed the prevalence of MDD in patients who have hyperparathyroidism (i.e. increased PTH by any cause). Hyperparathyroidism is a common endocrinological problem that can be found in patients with parathyroid adenoma, secondary and tertiary hyperparathyroidism, disorders of vitamin D deficiency, and chronic renal failure (that also includes dialysis patients), and disorders of calcium metabolism. Depression being a major prevalent neuropsychiatric symptom these days, we tried to find a relation between MDD and increased PTH levels.

We here wanted to see a relationship between depression and hyperparathyroidism. In that, we focused on mainly two types of studies; one type of study included pathophysiology of neuropsychiatric symptoms in the study material, and another type of study compared depression symptoms existence before and after surgery (parathyroidectomy) in their study material.

Pathophysiological Correlations Between Hyperparathyroidism and Diagnosed MDD

A study conducted by Wang and co-authors in the year 2021 showcased 38 patients with hyperparathyroidism who received a psychologic evaluation, through Beck Depression Inventory (BDI), State-Trait Anxiety Inventory (STAI), and 36-item Short Form Survey (SF-36) questionnaires out of which 32.2% of patients experienced anxiety, 31.8% were on the trait of anxiety, 18.4% of patients experienced mild to severe depression and 15.9% of patients had anxiety and depression both [2]. Serum calcium concentration (p = 0.006) was increased in patients with depression and anxiety. However, serum and urine cortisol concentrations had no statistical difference between the two groups (p > 0.05). A better study published in 2020 by Kunert and co-authors showed the coexistence of anxiety and depression in 101 patients with PHPT [1]. The study is better because it compared these patients with a control group of 50 patients suffering from non-toxic thyroid goiter. They assessed depression and anxiety scores through Hamilton Depression Rating Scale (HAM-D), the Beck Depression Inventory-II (BDI-II), and the Hospital Anxiety and Depression Scale (HADS). This study also compared depression symptoms in women and men in cohort and control groups. Assessing via HAM-D results observed in women were (p < 0.001) and results observed in men were (p > 0.05). The same results were found with BDI-II. A correlation between anxiety and depression in women with hyperparathyroidism (rs = - 0.2404; p < 0.05) was found but a correlation was not found for men. Also, the prevalence of anxiety and depression was found in patients with primary hyperparathyroidism and increased serum calcium levels (rs = - 0.1863; p < 0.05) if gender division was not done.

Duskin and co-authors in 2020 showed evidence of depression correlation in dialysis patients [10]. They assessed depression symptoms by kidney disease quality of life (KDQOL) form in 10 patients who had intact PTH values above 1000 pg/mL and in a control group of 10 patients who had PTH values less than 400 pg/mL and were going through dialysis for various diseases. The serum calcium is low or normal in secondary and tertiary hyperparathyroidism (common in dialysis patients). They wanted to test the correlation between PTH levels and depression in dialysis patients. They established that KDQL-38 was worse for patients with high PTH levels (p = 0.01). And they concluded more investigation targeting PTH and PTH2R (parathyroid hormone 2 receptors, in the brain) should be done to improve the quality of depressive symptoms in dialysis patients.

A case report of a 65-year-old male patient was reported by Sołtysik and co-authors in 2016 [16]. The patient was initially independent but subsequently developed neuropsychiatric manifestations and presented in the emergency department. The test for Creutzfeldt-Jacob disease was negative. The patient obtained 17 points on the Mini-Mental State Examination (MMSE). During inward treatment, additional blood tests revealed elevated alkaline phosphatase (ALP) 148 U/L (30-120), total calcium 3.74 mmol/L (2.20-2.65), and PTH level 74.46 pmol/l (1.6-6.9). No other abnormalities were found. After treatment, they concluded it was a case of PHPT in a geriatric patient with the main manifestation of severe but reversible neurocognitive syndrome. Also, a similar case report in 2012 by Borris and co-authors presented a geriatric case of a male aged 71 presented with apathy, significant weight loss, hyperoxia, sad mood (crying), and decreased water intake [15]. The study pointed out how less water intake, caused by depression, worsened hypercalcemia and showed a correlation between mental and organic pathology. They diagnosed him with depression related to hyperparathyroidism (particularly in this case because of hypercalcemia) and prescribed him escitalopram 10 mg daily.

These studies show a correlation between the pathophysiology of hyperparathyroidism (increased serum calcium level) and the patient’s neurocognitive behavior, particularly MDD. High serum calcium level in primary hyperparathyroidism patients was present with high depressive symptoms like sad mood, apathy, weight loss, etc. But depressive symptoms were not prominent with high serum and urine cortisol levels in primary hyperparathyroidism patients. Also, in a geriatric set of patients depressive symptoms and decreased water intake were noted with high parathyroid level, high serum calcium level, and high ALP levels, which was reversible once treated for hypercalcemia and their serum calcium and serum parathyroid levels were maintained to a normal level. In secondary and tertiary hyperparathyroidism serum calcium level remains normal or low, but depressive symptoms can be present with high serum parathyroid levels. 

**Figure 2:**
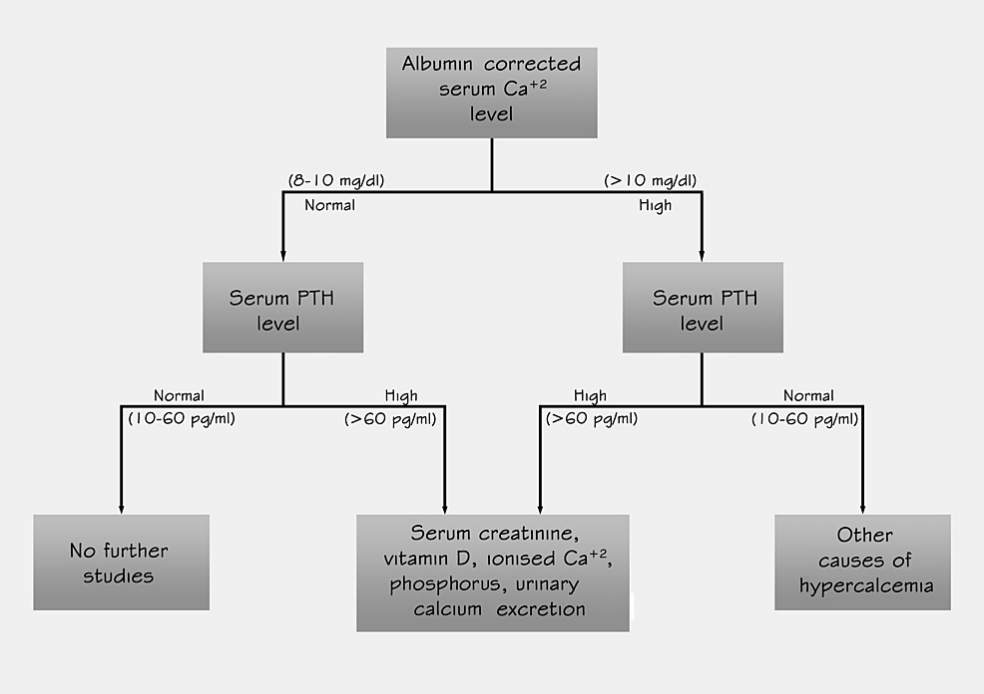
Biochemical examination procedure for patients with treatment-resistant depression, in order to detect whether a patient suffers from hyperparathyroidism The figure is adapted from reference [4] (PTH = parathyroid hormone, Ca+2 = calcium, mg/dl = milligrams per deciliter, pg/ml = picograms per milliliter, >= greater than)

Given flow chart (Figure 2) is created by corresponding authors adapted from a study by Grønli and co-authors published in 2013 [4]. Which outlines a biochemical examination procedure we can perform in patients with treatment-resistant depression, to detect whether a patient suffers from hyperparathyroidism or not.

Reversal of Depressive Symptoms After Treatment (Surgery) 

An observational study published by Lui and co-authors in 2021 included a total of 244 patients who underwent parathyroidectomy and a control group of 161 patients who underwent thyroidectomy [12]. The 244 patients had improved neuropsychiatric symptoms after parathyroidectomy (6.2 [5.0-7.4], p < 0.01). Before surgery, neuropsychiatric symptoms were more prevalent in patients undergoing parathyroidectomy compared to the patient undergoing thyroidectomy (11.2 ± 11.5 vs 7.5 ± 8.2, p < 0.01). They concluded 27.5% and 18.0% of patients before parathyroidectomy showed moderate to severe depression and anxiety, which decreased to 8.2% and 5.3%, after surgery. A similar study by Weber and co-authors (2013) presented decreased depression and anxiety in patients with parathyroidectomy after their 12-month follow-up of parathyroidectomy surgery; the prevalence of suicidal ideation was less than the baseline (10.7% vs 22%; p = 0.008) [13]. The 194 patients with PHPT represented their cohort and 186 patients with nontoxic thyroid nodules represented their control subjects. Both physical and mental health scores (45.7 and 47.7, respectively) were improved in the cohort (p < 0.001 each) but not in control subjects. The first study evaluated depression and anxiety in patients by Patient Health Questionnaire-9 (PHQ-9) and Generalized Anxiety Disorder-7 (GAD-7) to assess for depression and anxiety respectively. And the later study used HADS and PHQ-9, which also assessed suicidal ideation.

A study in 2014 by Hermsen and co-authors said that after parathyroidectomy changes in calcium and PTH in PHPT affect cortical excitability and improve subjective health [3]. They included 15 PHPT patients and measured cortical excitability via transcranial magnetic stimulation (TMS) which was altered before surgery. After surgery depression decreased (p = 0.05) though TMS, measurements did not change. Zanocco and co-authors in 2015 demonstrated similar outcomes in 35 patients with PHPT, with a control group of nine patients [11]. Patients were assessed by the Patient-Reported Outcomes Measurement Information System (PROMIS) score and the mean number of PROMIS items answered was 67 (range 51-121, SD 15.4). Improvement was noted in five domains of PROMIS in patients with the PHPT group after surgery but not in the control group, which included 8.8 in fatigue, 6.7 in sleep-related impairment, 5.0 in anxiety, 7.0 in applied cognition, and 6.2 in depression (all p < 0.05).

A case report about a woman was conducted in 2017 by Lopez and co-authors [15]. The patient experienced depressive episodes at ages 33, 53, and 62. She was suffering from bipolar disorder and had mixed presentations of manic and depressive episodes while admitted to the ward. On her laboratory results, she had severe hypercalcemia (corrected calcium 15.0 mg/dL), hypophosphatemia (2.4 mg/dL), and increased serum levels of PTH (983 pg/mL). Her cervical ultrasonography showed calcification in the left lower lobe of the parathyroid, parathyroid adenoma was suspected and was removed by surgery. After parathyroidectomy, her calcium levels decreased to 6.9 mg/dL. Postoperatively, a remarkable improvement in her mental health occurred. A similar case report was demonstrated by Grønli and co-authors in 2013 in which a 70-year-old man had suffered from prior four depressive episodes and was admitted to the psychiatric ward [4]. Where his serum calcium levels were normal but low serum albumin levels (28.6 g/L, normal range 36-45 g/L) and high PTH levels (9.3 pmol/L, normal range 1.1-7.5 pmol/L) were recorded. After his parathyroidectomy, his depressive symptoms subsided and he remained for three years without symptoms.

So, there is a relationship between increased parathyroid serum level and depressive symptoms and it vanishes or decreases after parathyroidectomy in patients with hyperparathyroidism. High depressive and anxiety symptoms are noted with high parathyroid levels in patients with PHPT which is decreased after parathyroidectomy and once low levels of parathyroid are established. If treated for high serum parathyroid levels there is a significant improvement in depressive symptoms. Parathyroidectomy will lower serum parathyroid level, lower serum calcium level, increase phosphorous level, and decrease serum ALP level. All effects combined decrease depressive and anxiety symptoms in the patient with hyperparathyroidism.

Limitations

Our systemic review included observational studies and case reports but we were lacking to find good randomized control trials for this paper. Two of the papers also did not have a control group which can be a drawback. We lack articles in all other languages except the English language.

## Conclusions

In conclusion, this systematic review suggests that major depressive disorder (MDD) is associated with hyperparathyroidism. There is much-proven disease prevalence in patients with hyperparathyroidism, psychosis being a major neuropsychiatric prevalence. But there are fewer papers discussing the association of depressive neuropsychiatric symptoms in patients suffering from hyperparathyroidism. This review weighs on the assessment of depressive symptoms in patients with increased parathyroid levels, so patients can be treated for their depressive neuropsychiatric symptoms if present, and physicians can help establish good mental and physical health in hyperparathyroidism patients via treating patients for their high parathyroid levels. We conclude that there is a major correlation in patients with hyperparathyroidism between high serum calcium level, high serum parathyroid hormone level, high serum alkaline phosphatase level, and depressive symptoms. In the end, we recommend that more studies should be done to check the pathophysiology of increased serum parathyroid levels and MDD neuropsychiatric symptoms. And more randomized control trials should be conducted to find the treatment effectiveness of depression in patients with hyperparathyroidism, so it can help improve mental health in patients suffering from hyperparathyroidism.
